# A phase I/II clinical trial for the hybrid of intracavitary and interstitial brachytherapy for locally advanced cervical cancer

**DOI:** 10.1186/s12885-016-2543-3

**Published:** 2016-08-17

**Authors:** Naoya Murakami, Shingo Kato, Takashi Nakano, Takashi Uno, Takeharu Yamanaka, Hideyuki Sakurai, Ryoichi Yoshimura, Junichi Hiratsuka, Yuki Kuroda, Kotaro Yoshio, Jun Itami

**Affiliations:** 1 Department of Radiation Oncology, National Cancer Center Hospital, 5-1-1 Tsukiji, Chuo-ku, Tokyo, 104-0045 Japan; 2 Department of Radiation Oncology, International Medical Center, Saitama Medical University, 1397-1 Yamane, Hidaka-shi, Saitama Japan; 3 Department of Radiation Oncology, Gunma University Graduate School of Medicine, 3-39-45 Showamachi, Maebashi, Gunma Japan; 4 Diagnostic Radiology and Radiation Oncology, Graduate School of Medicine, Chiba University, 1-8-1 Inohana, Chuo-ku, Chiba City, Chiba 260-8670 Japan; 5 Department of Biostatistics, Yokohama City University, 22-2 Seto, Kanazawa-ku, Yokohama, 236-0027 Japan; 6 Department of Radiation Oncology, University of Tsukuba, 1-1-1 Tennodai, Tsukuba, Ibaraki 305-8575 Japan; 7 Department of Radiation Therapeutics and Oncology, Tokyo Medical and Dental University, 5-45, Yushima 1-chome, Bunkyo-ku, Tokyo, 113-8519 Japan; 8 Department of Radiation Oncology, Kawasaki Medical School, Matsushima 577, Kurashiki, Japan; 9 Department of Radiation Oncology, Yamagata University Faculty of Medicine, Yamagata, 990-9585 Japan; 10 Department of Radiology, Kagawa Prefectural Central Hospital, 1-2-1 Asahi-cho, Takamatsu-shi, Kagawa Japan

**Keywords:** Uterine cervical cancer, Hybrid of intracavitary and interstitial brachytherapy, A prospective clinical trial protocol

## Abstract

**Background:**

This paper describes about a study protocol of phase I/II multicenter prospective clinical trial evaluating the feasibility and efficacy of the hybrid of intracavitary and interstitial brachytherapy (HBT) for locally advanced uterine cervical cancer patients.

**Methods and design:**

Patients with histologically confirmed FIGO stage IB2, IIA2, IIB, and IIIB uterine cervical carcinoma width of which is larger than 5 cm assessed by MRI will be entered to this clinical trial. Protocol therapy is 30-30.6 Gy in 15-17 fractions of whole pelvic radiotherapy concurrent with weekly CDDP (40 mg/m^2^), followed by 24 Gy in 4 fractions of HBT and central shield EBRT up to 50-50.4 Gy in 25-28 fractions. Tumor width is assessed again within one week before the first HBT and if the tumor width is larger than 4 cm, patients proceed to the secondary registration. In phase I section, feasibility of this will be investigated. If less than 10 % out of 20 patients experienced greater than grade 3 acute non-hematologic adverse effects, the study proceeds to phase II part. In phase II part a total of 55 patients will be accrued and the efficacy of the HBT will be investigated comparing with historical control data. If the lower margin of 90 % confidence interval of the 2-year pelvic progression-free survival of the HBT trial is higher than 64 %, the HBT is considered to be more effective than conventional ICBT.

**Discussion:**

The aim of this study is to demonstrate the feasibility and efficacy of the HBT for locally advanced cervical cancer. This trial will clarify the indication, feasibility, and efficacy of this new technique.

**Trial registration:**

UMIN000019081; Registration date: 2015/9/30

## Background

Standard primary radiation therapy for locally advanced cervical cancer is the combination of external beam radiation therapy (EBRT) and intracavitary brachytherapy (ICBT) with cisplatin based concurrent chemotherapy [1–5]. The classical ICBT [6] is based on the Manchester system [7, 8] or the Paris system [9], in which ICBT applicators consist of intrauterine (tandem) and vaginal source (ovoid or ring). This system has been used as the standard method for several decades. Although the Manchester method has been used for long time and favorable clinical results were reported so far [10–13], this system has a drawback; because the Manchester system was developed about a half century before, this system was based on two-dimensional, point-based system and used orthogonal x-ray images for dose calculation. The prescribed dose is delivered to a certain fixed reference point, point A, and this point is used independent of each tumor size or shape. Therefore, excellent tumor control can be expected for small tumors, while relatively high relapse rate was reported for large tumors part of which could not be covered by prescribed dose [14–16].

In locally advanced cervical cancer, tumors tend to spread laterally along with the cardinal ligament. Therefore, it was supposed to add a few interstitial needles in this region that would contribute to improve dose coverage and tumor control [17, 18]. Several positive clinical results have been reported concerning the hybrid of intracavitary and interstitial brachytherapy (HBT) for locally advanced cervical cancer [19, 20]. On the other hand, it was stated in the American Brachytherapy Society consensus guidelines that in a situation of poorly fitting intracavitary applicators, large lesions, and lower vaginal involvement interstitial brachytherapy (ISBT) should be considered [21]. There are overlaps of indication between ICBT alone, HBT, and ISBT alone and so far there exists no universal guideline for the indication of HBT and no prospective clinical trial focusing only on HBT for locally advanced cervical cancer.

The aim of this clinical trial is to investigate the feasibility, reproducibility, and efficacy of HBT for locally advanced cervical cancer who undergo primary chemoradiotherapy.

## Methods/design

### Study design

**Fig. 1:**
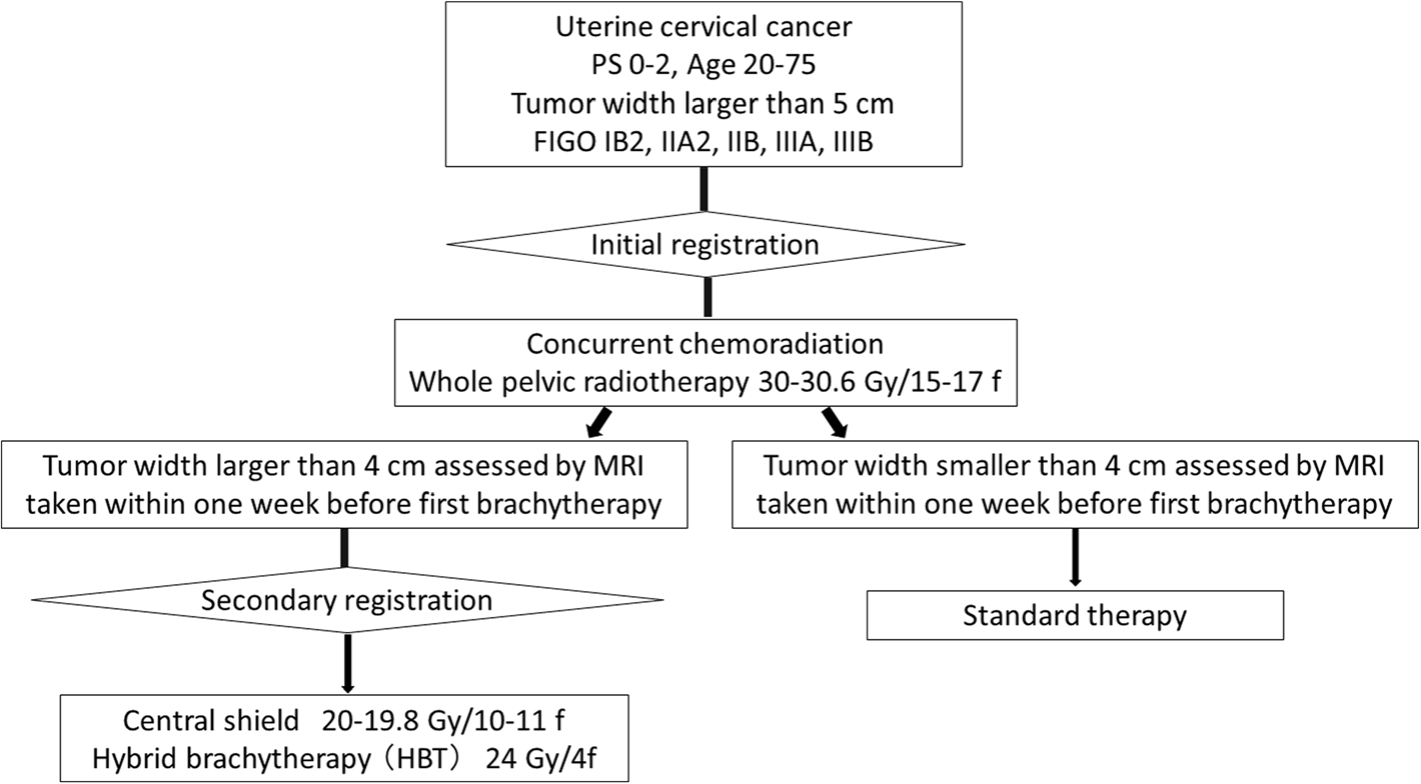
Study workflow

**Fig. 2:**
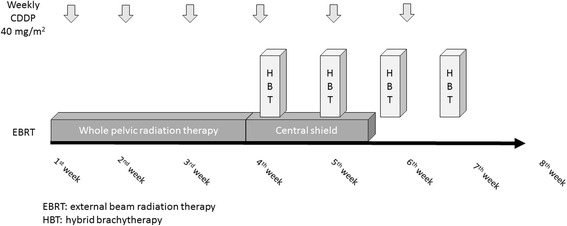
Overview of the protocol study

The HBT trial is a multi-institutional prospective phase I/II study. Figure 1 depicts the study workflow. Previously untreated patients with FIGO stage IB2, IIA2, IIB, and IIIB uterine cervical cancer with width of the tumor is larger than 5 cm assessed by MRI and who met the following inclusion criteria and who provided written informed consent proceeded to the initial registration. FIGO IVA disease is excluded because in FIGO IVA, tumor invades directly into adjacent rectum or bladder and additional interstitial needles into the rectum or the bladder are expected to cause more frequent acute non-hematologic adverse effects than other stages. Figure 2 shows overview of the protocol treatment. After receiving 30-30.6 Gy in 15-17 fractions of whole pelvic radiotherapy concurrent with weekly CDDP (40 mg/m^2^), 24 Gy in 4 fractions of HBT and central shield EBRT up to 50-50.4 Gy in 25-28 fractions are started. Tumor width is assessed within one week before the first HBT and in case of the tumor width is larger than 4 cm, patients proceeded to the secondary registration.

**Fig. 3:**
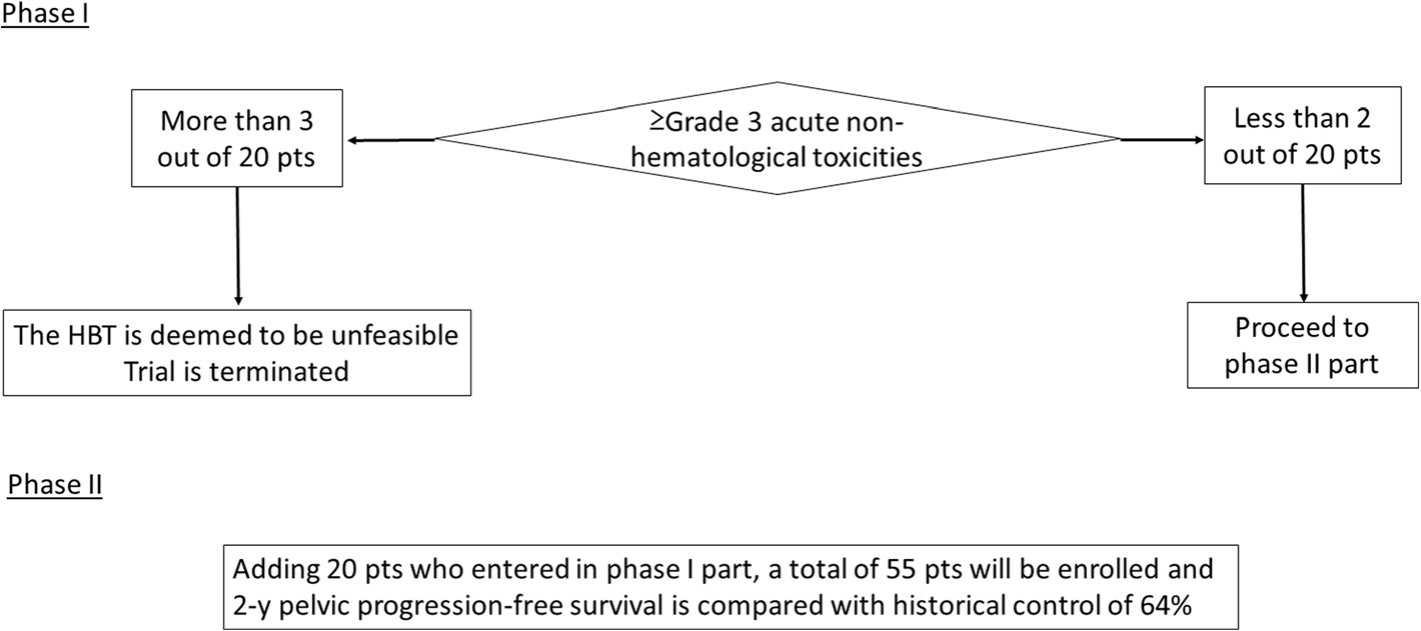
Stages of the study

Figure 3 shows the stages of the HBT study. First 20 patients who proceeded to the secondary registration will be enrolled in phase I part and the safety and tolerability of the HBT will be investigated. If less than 2 out of 20 patients (10 %) develop Grade 3 or higher acute non-hematological adverse effects, the HBT study proceeds to phase II part. In phase II part, 2-year pelvic progression-free survival is compared with historical control. Historical control data is cited from the publication of Pötter et al. [22] which demonstrated that 2-year pelvic progression-free survival rate of 64 % for patients with uterine cervical cancer whose initial tumor size was larger than 5 cm and were treated with conventional ICBT. Therefore, if the lower margin of 90 % confidence interval of the 2-year pelvic progression-free survival of the HBT trial is higher than 64 %, the HBT is considered to be more effective than conventional ICBT.

### Endpoints

The primary endpoint of this study in phase I part and in phase II part is the rate of acute non-hematologic adverse effects and the 2-year pelvic progression-free survival, respectively.

### Inclusion criteria

#### At initial registration

- Pathologically proven primary invasive uterine cervical carcinoma. Squamous cell carcinoma, adenocarcinoma, and adenosquamous cell carcinoma are eligible.
- Age between 20 and 75
- FIGO stage IB2, IIA2, IIB, IIIA, or IIIB
- Patients who will be treated with primary radiation therapy and who received no treatment including surgery, radiotherapy, or chemotherapy.
- Tumor width larger than 5 cm assessed by MRI taken within four week before the start of chemoradiotherapy.
- Eastern Cooperative Oncology Group (ECOG) Performance Status of 0-2
- Adequate organ function:
  - Hemoglobin > 8.0 g/dl
  - Neutrophils > 2000 cells/μl
  - Platelets > 50,000 cells/μl
  - Serum ALT/AST ≤ 100 IU/L
  - Serum Total bilirubin ≤ 1.5 mg/dL
  - Serum creatinine ≤ 1.2 mg/dL and creatinine clearance ≥ 60 ml/min
- No anticoagulant or antiplatelet medication
- No abnormal finding on electrocardiogram performed 14 days before study registration
- Written informed consent must be available before study registration

#### At secondary registration

- Initial registration is already done
- Tumor width is larger than 4 cm assessed by MRI taken within one week before initial session of HBT
- Eastern Cooperative Oncology Group (ECOG) Performance Status of 0-2
- White blood cell > 2000 cells/μl and platelets > 50,000 cells/μl

### Exclusion criteria

#### At initial registration

- Para-aortic lymph node metastasis with a short-axis diameter of greater than 10 mm assessed by CT or MRI
- Severe diabetes mellitus requiring continuous use of insulin
- Uncontrollable severe hypertension
- Unstable angina which occurred within three weeks or is recently exacerbating
- Transmural myocardial infarction within the last 6 months
- Simultaneous or metachronous (within 5 years) double cancers excluding carcinoma *in situ* or intramucosal tumor
- Active infectious disease to be treated
- Body temperature of 38 °C or more
- Psychiatric disease which hinders enrollment of clinical trial
- Active ulcerative colitis or Chron’s disease
- Active SLE or systemic sclerosis
- Allergy to local anesthesia
- Positive for HBs antigen

### At secondary registration

- FIGO IIIA disease the thickness of whose vaginal involvement exceeds 5 mm at the time at 30-30.6 Gy/15-17 fr and cannot be treated by ICBT alone
- Active infectious disease to be treated
- Body temperature of 38 °C or more

### Ethical aspects, trial registration

The HBT trial is approved by the institutional ethical review board of the National Cancer Center Hospital in accordance with the ethical standards laid down in the 1964 Declaration of Helsinki and its later amendments. The trial is registered with the UMIN (University Hospital Medical Information Network in Japan) Clinical Trials Registry, number UMIN000019081. Following is the list of participating centers where the study has received ethical approval: National Cancer Center Hospital, Yamagata University Faculty of Medicine, Kagawa Prefectural Central Hospital, Kawasaki Medical School, Tokyo Medical and Dental University, Graduate School of Medicine Chiba University, Institute of Health Biosciences the University of Tokushima Graduate School, Osaka Medical College, Research Center for Charged Particle Therapy National Institute of Radiological Sciences, Gunma University Graduate School of Medicine, National Hospital Organization Fukuyama Medical Center, Tokyo Rinkai Hospital, Tokyo Metropolitan Bokutoh Hospital, and Toyota Memorial Hospital.

### Therapy protocol

Figure 2 shows overview of protocol therapy. Weekly CDDP (40 mg/m^2^) is administered concurrently with EBRT. After 30-30.6 Gy in 15-17 fractions of whole pelvic radiotherapy, 24 Gy in 4 fractions of HBT and central shield EBRT up to 50-50.4 Gy in 25-28 fractions are started. If clinically swollen reginal pelvic lymph nodes exist, 6-10 Gy in 3-5 fractions of boost EBRT is performed.

### The HBT methods

**Fig. 4:**
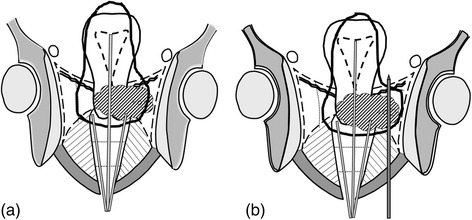
Schema of the concept of the hybrid brachytherapy (HBT). Figure 4a is a schema of conventional intracavitary brachytherapy (ICBT) in which tandem and ovoid are inserted in uterine cavity and vagina, respectively. Thick solid line represents isodose line of the prescribed dose. Tumor is represented by shaded structure which extends left parametrium and notice that left distal part of parametrium is not adequately covered by isodose line. Figure 4b is a schema of the HBT in which an additional interstitial needle is inserted to left parametrium covering of which is not enough with conventional ICBT. Notice that isodose line now covers whole tumor completely

Figure 4 demonstrates the concept of the HBT. Figure 4a is a schema of conventional ICBT. Thick solid line represents isodose line of the prescribed dose and tumor is represented by shaded structure which extends left parametrium. Left distal part of parametrium is not adequately covered by isodose line in Fig. 4a. Figure 4b is a schema of the HBT in which an additional interstitial needle is inserted to left parametrium and this additional needle can make isodose line cover the whole tumor completely. High-risk clinical target volume (HR-CTV) at HBT is delineated on CT taken with applicators in place. Because of limited availability of MRI, dose calculation of HBT is based of CT in this study. Definition of HR-CTV according to T stage is based on the contouring guideline proposed by Viswanathan et al. [23] with some modifications (Table 1). Table 2 summarizes dose constraints of HBT and the goal is to deliver more than 6 Gy to HR-CTV D_90_ (dose covering 90 % of the HR-CTV). In HBT, the diameter of hyper dose sleeve, which is the isodose line of 200 % of the prescribed dose, should be smaller than 1.5 cm and additional interstitial needles are restricted to three needles in one side and at most six needles in both sides. Tumors which cannot be covered with HBT based on these rules should be treated by ISBT alone with multiple interstitial needles.

**Table 1:** Definition of anatomical boundaries of high risk clinical target volume (HR-CTV) according to clinical stage

	Caudal margin	Cranial margin	Lateral margin	Posterior margin
IB	At superior level of the ovoid.	If uterine body involvement does not exist, upper limit of uterine cervix is cranial margin of HR-CTV for IB disease. As the surrogate structure of upper limit of uterine cervix, recognize the level at which uterine vessels first abut cervical tissue or to point at which uterine volume expands and uterine cavity appears. Add 8 mm around tandem superiorly to cover conical cervical apex. If direct uterine body involvement exists, measure the distance between fundus of the uterus and most cranial part of the tumor on MRI taken within one week before first brachytherapy. Subtract this distance from total length of the uterus and contour HR-CTV from the external os of the uterus until this subtracted length.	Width of HR-CTV is equal to that of uterine cervix.	-
IIA	Modify contour inferiorly to cover most inferior extent of vaginal extension using information derived from pelvic examination and MRI as a reference.	If uterine body involvement does not exist, upper limit of uterine cervix is cranial margin of HR-CTV for IB disease. As the surrogate structure of upper limit of uterine cervix, recognize the level at which uterine vessels first abut cervical tissue or to point at which uterine volume expands and uterine cavity appears. Add 8 mm around tandem superiorly to cover conical cervical apex. If direct uterine body involvement exists, measure the distance between fundus of the uterus and most cranial part of the tumor on MRI taken within one week before first brachytherapy. Subtract this distance from total length of the uterus and contour HR-CTV from the external os of the uterus until this subtracted length.	Width of HR-CTV is equal to that of uterine cervix.	-
IIB	If vaginal extension does not exists, contour until the superior level of the ovoid. If vaginal extension exists, modify contour inferiorly to cover most inferior extent of vaginal extension using information derived from pelvic examination and MRI as a reference.	If uterine body involvement does not exist, upper limit of uterine cervix is cranial margin of HR-CTV for IB disease. As the surrogate structure of upper limit of uterine cervix, recognize the level at which uterine vessels first abut cervical tissue or to point at which uterine volume expands and uterine cavity appears. Add 8 mm around tandem superiorly to cover conical cervical apex. If direct uterine body involvement exists, measure the distance between fundus of the uterus and most cranial part of the tumor on MRI taken within one week before first brachytherapy. Subtract this distance from total length of the uterus and contour HR-CTV from the external os of the uterus until this subtracted length.	Measure the width of tumor by the physical examination and/or trans-rectal ultrasonography (TRUS) and based on this length determine the width of HR-CTV on CT image. Determine the width of HR-CTV according to information of MRI taken before brachytherapy. If parametrial invasion is evident on CT image, rely on CT information. Caudal margin of parametrial invasion is set at superior level of the ovoid. Cranial margin of parametrial invasion is set at the cranial margin of cervix.	Contour HR-CTV posteriorly if uterosacral ligament invasion exists which is confirmed by pelvic examination, CT, or MRI.
IIIA	Contour HR-CTV so that the lowest extent of vaginal disease is adequately covered. Urethral meatus can be used as a anatomical landmark to compare CT, MRI, and physical examination.	If uterine body involvement does not exist, upper limit of uterine cervix is cranial margin of HR-CTV for IB disease. As the surrogate structure of upper limit of uterine cervix, recognize the level at which uterine vessels first abut cervical tissue or to point at which uterine volume expands and uterine cavity appears. Add 8 mm around tandem superiorly to cover conical cervical apex. If direct uterine body involvement exists, measure the distance between fundus of the uterus and most cranial part of the tumor on MRI taken within one week before first brachytherapy. Subtract this distance from total length of the uterus and contour HR-CTV from the external os of the uterus until this subtracted length.	If no parametrial involvement exists, contour until lateral edge of the uterine cervix. If parametrial involvement exists, refer to the description in IIB.	Contour HR-CTV posteriorly if uterosacral ligament invasion exists which is confirmed by pelvic examination, CT, or MRI.
IIIB	If vaginal extension does not exists, contour until the superior level of the ovoid. If invasion to upper 2/3 of vagina exists, refer to the description in IIA. If invasion to lower 1/3 to vagina exists, refer to the description in IIIA.	If uterine body involvement does not exist, upper limit of uterine cervix is cranial margin of HR-CTV for IB disease. As the surrogate structure of upper limit of uterine cervix, recognize the level at which uterine vessels first abut cervical tissue or to point at which uterine volume expands and uterine cavity appears. Add 8 mm around tandem superiorly to cover conical cervical apex. If direct uterine body involvement exists, measure the distance between fundus of the uterus and most cranial part of the tumor on MRI taken within one week before first brachytherapy. Subtract this distance from total length of the uterus and contour HR-CTV from the external os of the uterus until this subtracted length.	If parametrial involvement extends until pelvic wall, extend the lateral margin until pelvic wall such as inner margin of the obturator muscle or pelvic bone. If no parametrial involvement exists on contralateral side, refer to the description in IB. If parametrial involvement in contralateral side does not extend to pelvic wall, refer to the description in IIB.	Contour HR-CTV posteriorly if uterosacral ligament invasion exists which is confirmed by pelvic examination, CT, or MRI. If fixation of uterosacral ligament exists, extend HR-CTV to the sacral bone.

**Table 2:** Dose constraints for organ at risk (OAR)

OAR	Dose constraints for each HBT	Dose constraints for combination of EBRT and all HBTs (EQD2)
Rectum D2cc	<6.15 Gy	< 75 Gy
Bladder D2cc	< 7.30 Gy	< 90 Gy
Sigmoid D2cc	< 6.15 Gy	< 75 Gy

D2cc: most exposed 2 cc of tissue

EQD2: equivalent dose in 2 Gy fractions

### Statistics

#### Study hypothesis and sample size

In modern technique of image-guided ISBT for locally advanced uterine cervical cancer, the rate of grade 3 or higher acute non-hematologic adverse effects was reported to be 6.9 % [24]. In the HBT, less applicators are used compared to ISBT and dwell time of each applicator is supposed to be longer in the HBT than in the ISBT and complication is expected to be slightly higher in HBT than in the ISBT according to longer dwell time on each applicator. Therefore, threshold of the rate of grade 3 or higher acute non-hematologic adverse effects which are attributed to the HBT is set to be 10 % in this trial. In phase I part, 20 patients will be enrolled and if the rate of grade 3 or higher acute non-hematologic adverse effects happen in more than 3 patients (>10 %), the trial will be stopped.

In phase II part, it is hypothesized that HBT yields better 2-year pelvic progression-free survival rate than conventional ICBT for tumors which did not show good response to whole pelvic radiation therapy. Historical control data is cited from the publication of Pötter et al. [22] which demonstrated that 2-year pelvic progression-free survival rate of 64 % for patients with uterine cervical cancer whose initial tumor size was larger than 5 cm and were treated with conventional ICBT. If the lower margin of 90 % confidence interval of the 2-year pelvic progression-free survival of the HBT trial is higher than 64 %, the HBT is considered to be more effective than conventional ICBT. In phase II part, the planned sample size is 55 patients including 20 patients enrolled in phase I part, which was calculated based on an expected 2-year pelvic progression-free survival of 80 % and a threshold of 64.8 %, with a one-sided alpha error of 0.05 and a beta error of 0.2.

## Discussion

In the field of EBRT, there was a paradigm shift from two-dimensional to three-dimensional image-based treatment planning in last two decades. Likewise, in the field of brachytherapy, the same paradigm shift occurred from two-dimensional to three-dimensional image-based treatment planning. Currently the EMBRACE study (https://www.embracestudy.dk) [25] is running which investigates feasibility and efficacy of image-guided adaptive brachytherapy in multi-institutional setting, however, in the EMBRACE study both the ICBT and the HBT are allowed to be used without clear definition of which modality to adapt. There exists no prospective trial focusing only on the HBT. Therefore, this trial will elucidate the application, safety, and efficacy of the HBT and will make a cornerstone of the HBT for advanced uterine cervical cancer radiation therapy.

## Abbreviations

EBRT, external beam radiation therapy; ECOG, Eastern Cooperative Oncology Group; HBT, hybrid of intracavitary and interstitial brachytherapy; HR-CTV, high-risk clinical target volume; ICBT, intracavitary brachytherapy; ISBT, interstitial brachytherapy; UMIN, University Hospital Medical Information Network in Japan
